# The Longitudinal Association between Multiple Frailty Criteria and Depressive Symptomatology Five Years Later in Older Adults: A Decision Tree Modelling Approach

**DOI:** 10.3390/jcm13061697

**Published:** 2024-03-15

**Authors:** Marcelo de Maio Nascimento, Adilson Marques, Élvio R. Gouveia, Priscila Marconcin, Miguel Peralta, Andreas Ihle

**Affiliations:** 1 Department of Physical Education, Federal University of Vale do São Francisco, Petrolina 56304-205, Brazil; 2 Swiss Center of Expertise in Life Course Research LIVES, 1227 Carouge, Switzerland; andreas.ihle@unige.ch; 3 CIPER, Faculty of Human Kinetics, University of Lisbon, 1649-004 Lisbon, Portugal; amarques@fmh.ulisboa.pt (A.M.); mperalta@fmh.ulisboa.pt (M.P.); 4 ISAMB, Faculty of Medicine, University of Lisbon, 1649-004 Lisbon, Portugal; 5 Department of Physical Education and Sport, University of Madeira, 9000-082 Funchal, Portugal; erubiog@staff.uma.pt; 6 Laboratory of Robotics and Engineering Systems (LARSYS), Interactive Technologies Institute, 9000-082 Funchal, Portugal; 7 Center for the Interdisciplinary Study of Gerontology and Vulnerability, University of Geneva, 1227 Carouge, Switzerland; 8 Insight: Piaget Research Center for Ecological Human Development, 1950-157 Lisbon, Portugal; priscila.marconcin@ipiaget.pt; 9 Faculty of Health Sciences, Universidad Autónoma de Chile, Providencia 3460000, Chile; 10 Department of Psychology, University of Geneva, 1227 Carouge, Switzerland

**Keywords:** ageing, frailty, mental health, decision tree, SHARE database

## Abstract

**Background**: To examine the longitudinal association between frailty criteria and depression (DEP) in a large sample of older Europeans using decision tree models, and to examine complex relationships between frailty criteria and DEP symptomatology. **Methods**: Data come from waves six and eight of the Population Survey of Health, Ageing and Retirement in Europe. DEP was assessed using the EURO-D scale (wave 8) and frailty (wave 6). We included 27,122 people (56.9% women), aged 50 or over. **Results**: Women indicated a higher rate of DEP (29.0%), as well as a higher prevalence of pre-frailty (21.6%) and frailty (10.8%) than men. For both sexes, fatigue, weight loss, and slowness indicated an increased chance of DEP 5 years later. MPA (moderate physical activity) and grip strength were considered longitudinally protective factors for DEP. The highest prevalence of DEP symptomatology 5 years later was 50.3%, pointing to those with fatigue and slowness. Among women, the highest incidence of DEP was 66.8%, identified through fatigue, slowness, and low MPA. **Conclusions**: Strategies to reduce frailty and DEP in older European adults may include the creation of policies that encourage the promotion of physical capacity to reach MPA levels, as well as an improvement in muscular strength.

## 1. Introduction

The ageing process generates physiological changes that gradually affect several body systems [1,2], impairing the individual’s physical, cognitive, and emotional functions [3,4]. Frailty and depression (DEP) are two common conditions of ageing. If not identified and treated early, they can evolve and cause serious health problems. Previous studies have reported an association between both [5,6]. Frailty is a stress response to multisystem dysregulation of the musculoskeletal and metabolic systems, generating reduced resilience [5]. In turn, the same stress responses are also present in late DEP [7]. DEP symptomatology is a disease with serious consequences; due to this, the World Health Organization has recognized it as the third main contributor to the global burden of disease [8].

DEP is considered a common disease; however, it has serious consequences, capable of affecting both mental and physical health [9]. In 2015, depressive disorders led to a global total of more than 50 million years lived with disability (YLD) [8]. It is estimated that by 2030, in developed countries, DEP symptomatology will reach first place among the most common diseases in the population [10]. Late DEP symptomatology is a silent disease that can remain masked due to associated factors [7,8]. Its causes may result from dysfunctions of specific pathophysiological mechanisms such as neural hormones and immunometabolic issues [11,12]. Among the symptomatology, there is a loss of interest and pleasure in common experiences, a lack of interest in social exchanges, depressed mood, and deficits in cognitive and physical functions.

In turn, the frailty phenotype is a biological syndrome of diminished reserve and resistance to stressors, resulting from cumulative declines in various physiological systems and causing vulnerability to adverse outcomes [13]. This definition includes events of falls, hospitalizations, and disabilities, in addition to death resulting from frailty. The criteria for a physical frailty phenotype are reduced physical activity (PA) level, unintentional weight loss, slowness, low muscle strength, and exhaustion [14]. Moreover, among older adults, the combination of chronic diseases and sedentary behavior intensifies physical frailty [15], affecting functional independence and emotional state. When it comes to frailty and DEP, the overlapping spectrum of both is a challenge for the early diagnosis of DEP symptomatology [6,11], making it difficult to initiate adequate treatment. 

In countries with ageing populations, such as Europe, the prevalence of DEP is high [16]. The FRALLE study (Spain) (≥75 years) indicated that DEP symptomatology was associated with greater odds of frailty (OR = 3.13; 95% CI = 1.37–7.13) [17]. In Italy, as part of the InCHIANTI study (≥65 years), follow-ups at 3, 6, and 9 years showed that, among frail older adults, 30.6% of those who were not depressed developed a depressed mood [18]. Consequently, frailty generated a risk of triggering a new DEP event (HR = 1.26; 95% CI = 1.09–1.45). A recent 5-year longitudinal study that investigated the association between frailty and DEP in older adults from 17 European countries pointed to individuals who, in 2015, presented the condition of pre-frailty and frailty, with odds of 1.86 (CI 95% 1.71, 2.01) and 2.46 (95% CI 2.14, 2.83) to present DEP symptomatology in 2020 [19].

Although these studies have expanded the understanding of the relationship between frailty and DEP in the older European population, the results differ substantially. The literature highlighted no consensus on the prevalence of DEP in the older European population [20,21]. One explanation is that DEP rates depend on factors specific to each population, such as health contexts, social, economic, and cultural issues, and religious beliefs [22,23]. In turn, there are still gaps regarding the specific role that each frailty criterion and the type of frailty (e.g., pre-frail, frail) contributes to the establishment of DEP symptomatology over the years. 

Another issue to be explained is the bidirectional relationship between DEP and frailty according to sex. When it comes to DEP, there is no consensus on its prevalence in relation to sex. For instance, some studies observed worse symptomatology among women [24,25], while others found no difference between the sexes [26]. However, though there are suspicions that the prevalence of DEP symptomatology is high among men, underreporting occurs due to impediments related to masculinity [27]. Sex differences were also found in frailty, which is prevalent among women [28]. A meta-analysis study revealed an interesting issue [29]. Although frailty is greater among women, they were more tolerant than men to adverse health factors; regardless of age group or level of frailty, women had a lower mortality rate. The findings suggested that there is a health-survival paradox in relation to the sexes.

In addition, the spectrum of cultural, social, economic, and political differences between the populations of European countries is large, making it difficult to have an integrated understanding of the physical and mental health of populations [30,31]. Thus, it is important to develop longitudinal investigations focusing on the relationship between DEP and frailty based on large populations. Therefore, this study aimed to examine the longitudinal association between multiple frailty criteria and DEP symptomatology five years later in a large sample of older adults (50 years and older) from 12 European countries and to examine, using decision tree models, complex relationships between frailty criteria and DEP symptomatology.

The decision tree is a common statistical procedure in public health investigations, functioning as a predictive model to conclude a set of observations [32]. This analysis has been used to examine DEP in adult populations, focusing on environmental, social, and financial factors [33,34]. To our knowledge, this approach has not yet been applied to determine the weight each frailty criterion can add to establishing DEP symptomatology. Thus, considering that the combination of frailty and DEP symptomatology in the older population leads to an increased risk of adverse outcomes, including mortality [23], our results may be useful in the early identification of the main frailty criteria responsible for the development of DEP symptomatology. In turn, the finds can complement current public aging policies in several European countries.

## 2. Materials and Methods

### 2.1. Study Design and Participants

This longitudinal, cross-national, population-based study used data from the Survey of Health, Ageing and Retirement in Europe (SHARE) [35]. SHARE studies the effects of health, social, and environmental policies since 2004 in 28 European countries, including Israel. For the present study, we selected waves 6 (year 2015) and 8 (year 2020). The sample included 27,122 respondents, aged 50 and over (11,688 men; 15,434 women). Participants came from twelve countries: Austria, Belgium, Czech Republic, Denmark, France, Germany, Greece, Italy, Poland, Spain, Sweden, and Switzerland. Data were collected through face-to-face interviews in participants’ homes using computer-assisted personal interviews (CAPI). All participants provided consent before the interviews. The SHARE protocol was reviewed and approved by the Ethics Committee of the University of Mannheim and the Ethics Committee of the Max-Planck Society for the Advancement of Science. All procedures followed the guidelines and ethical standards of the Declaration of Helsinki.

### 2.2. Measures

#### Dependent Variable: Depression

DEP was measured using the Depression Symptoms Scale of the EURO-DEP consortium (EURO-D scale) [36]. The EURO-D scale has 12 items that investigate: depressed mood, pessimism, death wish, guilt, sleep, interest, irritability, appetite, fatigue, concentration, enjoyment, and tearfulness. The item responses have a score of 0 (symptomatology absent) or 1 (symptomatology present), resulting in a score ranging from 0 to 12. Thus, the higher the total score, the greater the indication of depressive symptomatology. The cutoff point used to screen for depressive symptomatology was ≥4 points. The scale presents acceptable reliability, with Cronbach’s alpha ranging from 0.71 to 0.72 across waves [36]. We used data from SHARE wave 8 (2020) for analysis.

### 2.3. Independent Variables 

#### Frailty

Frailty was assessed using the SHARE-Frailty (SHARE-FI) instrument [37]. The SHARE-FI assessed five constructs of Fried’s frailty phenotype [14]. Of these, four were self-reported (i.e., fatigue, weight loss, moderate physical activity (MPA), slowness), while muscle weakness was assessed by handgrip strength, using a portable dynamometer (Smedley, S Dynamometer, TTM, Tokyo, Japan, 100 kg). The variables fatigue, weight loss, and slowness were computed in the self-report as binary (yes/no). The MPA variable was computed based on three responses: (1) MPA < once per week, (2) MPA once per week, and (3) MPA > once per week. Grip strength measurements with values of 0 kg or ≥100 kg were excluded, as were measurements recorded with only one hand. The grip strength cutoff points (Kg) assumed as a frailty criterion were those suggested by Fried [14], calculated depending on the body mass index (BMI): (1) women (BMI ≤ 23: ≤17 kg; BMI 23.1–26: ≤17.3 kg; 26.1–29: ≤18 kg; >29 kg: ≤21 kg); (2) men (BMI ≤ 24: ≤29 kg; BMI 24.1–26: ≤30 kg; BMI 26.1–28: ≤30 kg; BMI > 28: ≤32 kg). In turn, frailty classification was established individually for men and women using a composite frailty scoring algorithm based on the five assessments. In turn, the classification of study participants was categorized according to a previous study using the following criteria [37]: non-frail (0 criteria present), pre-frail (1 or 2 criteria present), or frail (≥3 criteria). Data from SHARE wave 6 (year 2015) were used for these analyses.

### 2.4. Covariates

Six self-reported variables were considered in the analysis as confounding factors (i.e., age, education, type of housing [lives/does not live with a partner], body mass index (BMI), comorbidities, and disability). The level of education was established based on the International Standard Classification of Education (ISCED) [38]. The different levels were aggregated into three categories: (1) ISCED 0–1: no education or a low level of education, (2) ISCED 2–4: intermediate level of education, and (3) ISCED 5–6: a higher level of education. Participants reported their height and weight, and BMI was calculated (weight in kg/m^2^). BMI was categorized as underweight (<18.5), normal weight (18.5 to 24.9), overweight (25 to 29.9), or obese (≥30) [39]. The number of chronic diseases indicated comorbidities. The information was based on a medical report (last 12 months). This measure is reliable for categorizing general health status [40]. An individual score was established through two categorizations: ≤2 or >2 chronic diseases. Disability was obtained through self-reporting one or more difficulties in carrying out activities of daily living, such as impediment to going to the bathroom, bathing, dressing, eating, walking, getting in/out of bed (binary measurement). Data from wave 6 (year 2019) was used for the analyses.

### 2.5. Statistical Analyses

Initially, data normality was checked using the Kolmogorov-Smirnov test. The stratification of the sample was done by sex. Differences between men and women were calculated using the Chi-square test (categorical variables) or the Mann-Whitney U test (metric variables). The variables fatigue, weight loss, and slowness (yes/no) were presented as percentages, as were the three categories of MPA responses. Furthermore, using mean and standard deviation, a sum of the MPA categories for each group was presented. Using univariate and multivariate binomial logistic regression analyses, the association of the independent variable frailty was verified (i.e., fatigue, weight loss, slowness, MPA, grip strength) with the dependent variable DEP (yes/no). Preceding the analyses, we inspected the variance inflation factor (VIF) to check for multicollinearity in the multivariate model. Results were presented by odds ratio (OR) and their respective confidence intervals (95% CI). For this analysis, all independent variables were entered simultaneously, resulting in two different models: Model 1, which was unadjusted, and Model 2, which was adjusted, for covariates (i.e., age, type of housing, BMI, comorbidities). 

Finally, using decision tree analysis, we longitudinally examined data on how the factors responsible for frailty (x), wave 6, acted on DEP (y), wave 8. Decision trees are an analytical technique based on machine learning [41]. In this way, through various classes of modelling algorithms, similar subjects are grouped and classified in a hierarchical scale of nodes in the structure of a tree. The methodology has previously been successfully applied in public health in disease screening and prediction services [42], and in studies on DEP symptomatology [33,34]. Two models were calculated: one for men and one for women. The method is appropriate for classifying and predicting the target variable (DEP), offering the rules that classify the target object into several subgroups through the structure of a tree [43]. Moreover, the analysis calculates interactions between continuous variables and discrete variables [44]. We used the automatic Chi-square interaction detection method (CHAID) as the basis for separation (growth) and CART (Classification and Regression Trees). The CART method is suitable for predicting dichotomous variables by conducting multiple divisions by checking the Chi-square. 

In practice, the basic structure of a CHART is composed of nodes [44]: (1) The main node or root node, also called the decision node, represents a choice that will lead to independent divisions of the other data into two or more subsets; (2) Internal nodes or chance nodes, which are one of the possible decisions at a given point in the tree structure (the top part of the node is connected to its parent node, while the bottom part is connected to child nodes or leaf nodes); and (3) Leaf nodes, which represent the result of a combination of decisions or events. A set of internal nodes and leaf nodes is established according to an influence pattern determined by the specific weights of each processed data. A tree depth of four levels was assumed for the stopping rules (e.g., segment formation). Therefore, the minimum number of parent node and child node cases was set to 100 and 50, respectively. Our analysis also included profit and risk graphs. Using this resource, it was possible to generate information about the adjustment of each model. The gains graph presents the percentage of the target category (DEP symptomatology) in each node generated by the independent variable (frailty factors).

The literature highlighted that temporal analyses can be influenced by reverse causality bias [45], which would be caused by a previous presence of DEP symptomatology in the present study. Therefore, we performed analyses to control for possible bias in the baseline. When comparing the results, we verified a similar pattern between the outcomes with and without exclusion of participants with DEP symptomatology. Due to this, we chose not to exclude, at the beginning of the study, those with a previous diagnosis of DEP symptomatology. All statistical analyses were performed using SPSS version 24. The significance level was set at *p* < 0.05.

## 3. Results

### 3.1. Baseline Characteristics

The baseline characteristics of the participants (2015) and the EURO-D scale score and frailty criteria are presented in Table 1. Men had a slightly higher mean age, higher BMI than women, and a higher prevalence for intermediate to higher levels of education (*p* < 0.001). Women had a higher number of comorbidities and obesity (*p* < 0.001), while men had a higher prevalence of being overweight (*p* < 0.001). In turn, men indicated greater years of education and greater tendency to live with a partner (*p* < 0.001). Comparatively, women showed, on average, more symptomatology for DEP than men (2.57 ± 2.20 vs. 1.75 ± 1.84, *p* < 0.001), as well as a higher prevalence of pre-frail (21.6% vs. 8.9, *p* < 0.001) and frail (10.8 vs. 1.6, *p* < 0.001) condition.

### 3.2. Binomial Regression Analysis

The 5-year longitudinal association between frailty (wave 6) and DEP (wave 8) according to sex is presented in Table 2. Regarding men, the unadjusted model was statistically significant [X^2^(5)= 899.17; *p* < 0.001, R^2^ _Nagelkerke_= 0.12]. DEP was positively associated with slowness, weight loss, and fatigue (*p* < 0.001), indicating a chance of increasing DEP symptomatology by up to 53%, 44.2%, and 65.4%, respectively. In turn, MPA and grip strength indicated a negative association with DEP (*p* < 0.001), acting as protective factors, with a chance of reducing DEP by up to 0.09% and 0.02%, respectively. After adjusting for covariates (i.e., age, education, BMI, comorbidities, disability), the model remained significant [X^2^(4)= 88.11; *p* < 0.001, R^2^ _Nagelkerke_= 0.26]. DEP was positively associated with slowness, weight loss, and fatigue (*p* < 0.001), representing an increase in the chance of DEP by up to 44.8%, 41.8%, and 63.1%, respectively. The associations of MPA and grip strength with DEP were negative (*p* < 0.001), with the chance of DEP reduction being approximately 0.09% and 0.02%, respectively.

Regarding women (Table 2), the unadjusted model was significant [X^2^ (5) = 1491.03; *p* < 0.001, R^2^ _Nagelkerke_= 0.13]. Slowness, weight loss, and fatigue were positively associated with DEP (*p* < 0.001), indicating an increase in the chance of presenting DEP symptomatology by up to 43.8%, 42.2%, and 60.9%, respectively. On the other hand, MPA and grip strength revealed a negative association with DEP (*p* < 0.001), indicating a reduction in the chance of DEP of approximately 14.4% and 0.03%, respectively. After adjusting for covariates (i.e., age, education, BMI, comorbidities, disability), the model remained significant [X^2^(4)= 198.84; *p* < 0.001, R^2^ _Nagelkerke_= 0.26]. Slowness, weight loss, and fatigue were positively associated with DEP (*p* < 0.001), which showed a chance of increasing DEP by up to 31.0%, 41.2%, and 57.9%, respectively. The associations of MPA and grip strength were negative (*p* < 0.001), representing a chance of DEP reduction of approximately 12.8% and 0.3%, respectively.

### 3.3. Results for Men

**Figure 1:**
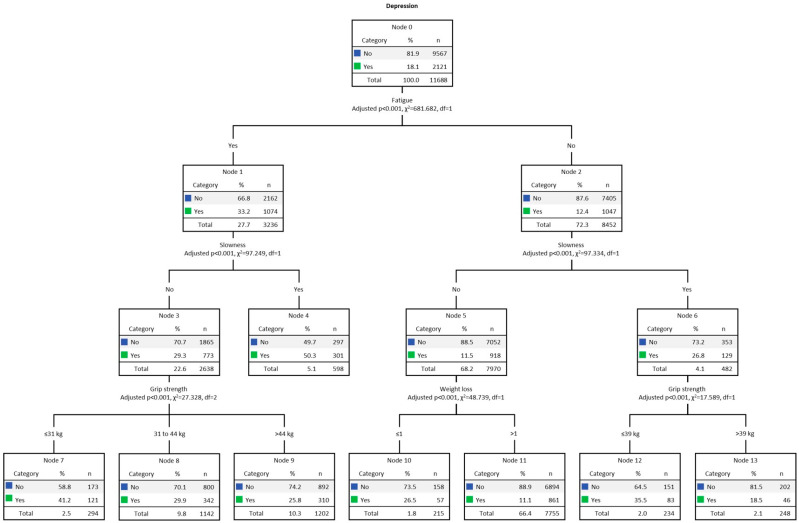
Decision tree analysis (CHAID) for men’s DEP. Note: weight loss ≤ 1, no; weight loss > 1, yes.

Figure 1 presents the multivariate decision tree analysis (CHAID) for men’s DEP, adjusted for the five frailty criteria. The overall DEP rate among men was 18.1% (n = 2121). The final model was formed by 3 levels of depth and 14 nodes. The strongest predictor of DEP was fatigue (Chi-square = 681.68, *p* < 0.001). The DEP rate for men with fatigue (Node 1) was 33.2% (n = 1074). In turn, in the second line, the strongest predictor for DEP among those who presented fatigue was slowness (Chi-square = 97.25, *p* < 0.001), and DEP was present in 50.3% (n = 301, Node 4). On the other hand, among men without slowness (Node 3), DEP was present in 29.3% (n = 773). In the third row, the strongest predictor of DEP was grip strength (Chi-square = 27.33, *p* < 0.001). Consequently, it was found that reduced grip strength, present in 41.2% (n = 121), indicated the highest DEP index (Node 7). On the other side of the tree (Node 2) we find those without fatigue, present in 12.4% (n = 1047). In the second line, the strongest predictor of DEP was also slowness (Chi-square = 97.34, *p* < 0.001), present in 26.8% (n = 129, Node 6). In the third row, the strongest predictor of DEP was grip strength (Chi-square = 17.59, *p* < 0.001). DEP was present in 35.5% (n = 83) of those with low grip strength index (Node 12). Among those without slowness (Node 5), present in 11.5% (n = 918), on the third line, the strongest predictor of DEP was weight loss (Chi-square = 48.74, *p* < 0.001). DEP was prevalent in 26.5% (n = 57) of men with weight loss (Node 10), an indication of malnutrition and disability.

Table 3 presents the gain results. Data shows the predictive power of each model for the target category. The gain score by the node is displayed in descending order. The node with the highest gain score was number 4 (i.e., men with fatigue and slowness). Node 4 computed 598 individuals, 5.1% of the total sample (n = 11,688). The number of cases (gain: n) corresponding to the target category was 301, representing 50.3% (response %) of the total cases. In turn, the node index was 277.4%. Finally, the decision tree correctly classified 82% of the cases (risk estimate = 0.18; SE = 0.004), indicating a good fit. 

### 3.4. Results for Women

**Figure 2:**
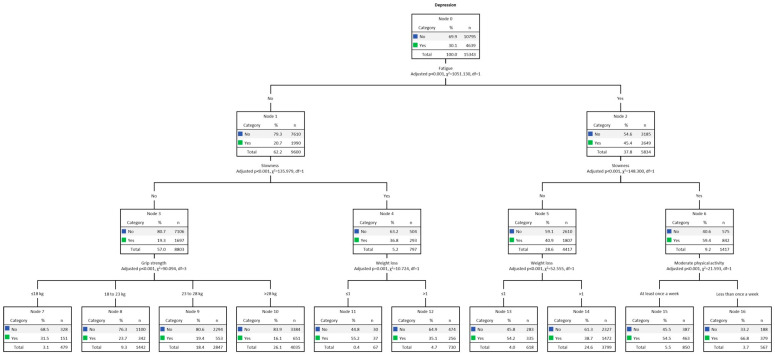
Decision tree analysis (CHAID) for women’s DEP. Note: weight loss ≤ 1, no; weight loss > 1, yes.

Multivariate decision tree analysis (CHAID) for DEP among women, adjusted for the five frailty criteria, is shown in Figure 2. The overall DEP rate among women was 30.1% (n = 4639). The final model was formed by 3 levels of depth and 14 nodes. The strongest predictor of DEP was fatigue (Chi-square = 1051.13, *p* < 0.001). The DEP rate for women with fatigue (Node 2) was 45.4% (n = 2649). The second strongest predictor for DEP among those who experienced fatigue was slowness (Chi-square = 148.30, *p* < 0.001), where DEP was present in 59.4% (n = 842) of women (Node 6). Therefore, the third strongest predictor for DEP was MPA (Chi-square = 21.59, *p* < 0.001), present in 66.8% (n = 379) of those with MPA less than once a week (Node 16). On the other hand, among women for whom slowness was not present (Node 5), 40.9% (n = 1807), the third strongest predictor for DEP was weight loss (Chi-square = 52.56, *p* < 0.001), present in 54.2% (n = 335) (Node 13). On the other side of the tree, at Node 1, responsible for women without fatigue, the DEP rate was 20.7% (n = 1990). In the second row, the strongest predictor was slowness (Chi-square = 135.98, *p* < 0.001). Among those with slowness (Node 4), DEP was present in 36.8% (n = 293). In the third row, the strongest predictor was weight loss (Chi-square = 10.72, *p* < 0.001). The highest prevalence of DEP occurred among those with weight loss (Node 11), present in 55.2% (n = 37). Among women without fatigue and slowness (Node 3), DEP was present in 19.3% (n = 1697). Therefore, the third strongest predictor of DEP was grip strength (Chi-square = 90.09, *p* < 0.001), and DEP was prevalent in those with low performance (Node 7), present in 31.5% (n = 151).

Table 4 presents the gain results. The highest gain score was indicated by Node 16 (i.e., women with fatigue, slowness, and low MPA). Node 16 computed 567 individuals, 3.7% of the total sample (n = 15,434). The number of cases (gain: n) corresponding to the target category was 379, which means 66.8% (response %) of the total number of cases. In turn, the node index was 222.4%. Finally, the decision tree correctly classified 72% of the cases (risk estimate = 0.28; SE = 0.004), indicating a good fit.

## 4. Discussion

This study examined the longitudinal association between multiple frailty criteria and DEP five years later in a large sample of older adults (50 years and older) from 12 European countries. Moreover, it aimed to examine, using decision tree models, complex relationships between frailty criteria and DEP symptomatology. Generally, women indicated a higher incidence of DEP symptomatology than men and a higher prevalence for the pre-frail and frail condition. Our results were in line with a recent 5-year longitudinal study carried out with the older population of 17 countries in the SHARE database [46]. Therefore, the unadjusted and adjusted analysis for confounders (i.e., age, education, BMI, comorbidities, disability) revealed fatigue, weight loss, and slowness as responsible for the increased chance of DEP symptomatology 5 years later for both sexes. On the other hand, in both analyses, MPA and grip strength criteria proved to be longitudinally protective factors against DEP for men and women. 

Regarding the decision tree analysis, for both sexes, the fatigue and slowness criteria were the first and second strongest predictors of DEP symptomatology, respectively. An interesting finding of the present study was that, for both men and women, decision tree procedures confirmed the results of the regression analysis, pointing to the fatigue and slowness criteria as the first and second strongest predictors of DEP symptomatology 5 years later. Thus, while among men presenting fatigue and slowness there was a higher prevalence for DEP (50.3%) 5 years later, among women, fatigue and slowness still included a low level of MPA as responsible for the highest prevalence of DEP 5 years later (66.8%). According to gains analysis, decision trees correctly classified 82% of the cases of men and 72% of the cases of women. Moreover, the node index for both sexes was greater than 200%. There is no clear pattern for the win rate, but if a specific node’s rate exceeds 200%, the winning score will be very high [47,48].

A study presented by the SHARE group highlighted that, although European women lived longer than men, on the other hand, they indicated a worse state of health [49]. A possible explanation for the paradox between the sexes is that women naturally develop more comorbidities over the years than men, which may be related to genetic patterns. On the other hand, men are at an increased risk of developing fatal diseases (i.e., heart disease and stroke) resulting from behavioral health patterns, such as a less healthy diet and greater consumption of tobacco and alcohol [50]. Thus, when it comes to frailty, the literature suggested a series of sexual differences conditioned by biological factors, such as sarcopenia [51], body composition [52], behavioral healthcare utilization [53], and cognitive frailty [54]. It is not excluded that there are bidirectional causal relationships between frailty and DEP symptomatology, since both frail individuals and those who are depressed may have low reserves of physical energy and lack of interest in carrying out basic daily life tasks, as well as a lack of desire to interact socially [55]. Consequently, the presence of a combination of frailty criteria (i.e., fatigue, weight loss, MPA, slowness) can trigger emotional problems (i.e., loss of mood, sadness, loneliness, anxiety, stress, fear, insomnia), leading the individual to a depressive state [56,57]. Therefore, the association between frailty and DEP symptoms is a rapid mechanism capable of making older adults more vulnerable, increasing morbidity and mortality [58].

Another factor common to frailty and DEP with specific sex differences are the levels of inflammatory cytokines [59,60]. DEP and fatigue are linked to increased inflammatory activation of the immune system, affecting the central nervous system [61,62]. A current overview that includes 43 meta-analyses to verify factors related to inflammation and depressive symptomatology suggested inflammation as a suitable biomarker to identify psychiatric disorders [63]. This demonstrates the bi-directionality between inflammation and neuropsychiatric disorders [64]. This can also be attributed to depression, which potentiates inflammatory reactions [65]. In this way, an arrangement is established in the human organism between a series of factors that both facilitate and accelerate a state of fragility. 

Our decision tree analysis confirmed and extended the longitudinal outcomes presented by the regression analysis. Among men, while the combination of fatigue and slowness was revealed as the highest prevalence of DEP (second line of the tree), in the group of women, the strongest predictor of DEP was indicated in the third line (i.e., low level of MPA). A possible explanation for this may lie in the combination of the characteristics of the members of both groups, verified in the baseline (year 2015). Therefore, although, comparatively, the average MPA results for both sexes were slightly higher for men, when it came to fatigue and slowness, women indicated a higher rate. Furthermore, the EURO-D scale showed that women were more depressive and reported a greater number of comorbidities in the range of 4–9 types. In turn, a greater arrangement between comorbidities, fatigue, and DEP present in women suggests the existence of inflammatory processes [65,66].

An interesting finding of this study occurred in the third line of the women’s tree, indicating a low MPA level as the strongest predictor of DEP. This information serves as a warning because if low levels of PA harm mental health [67], on the other hand, high levels of PA can neutralize a series of associated factors responsible for a higher risk of frailty and DEP [68,69]. Over the years, studies have shown that sedentary lifestyles can be reversed at an advanced age [70,71]. Thus, increasing caloric expenditure, for example, through the regular practice of physical exercises, is considered effective for older adults to reach adequate daily/weekly levels of MPA, or even a vigorous PA level [72,73]. Our findings also corroborate a recent study carried out with older adults [74] which found, in both sexes, a negative association between DEP symptomatology and moderate and high levels of PA. 

Aging is also associated with a progressive decline in skeletal muscle mass and strength. This process is called sarcopenia, which leads to a loss of muscle strength and, consequently, to the development of physical disability [75]. The case is even more acute in women after menopause [76]. On the other hand, in advanced age, an increase in PA levels, especially regular physical exercise, can increase grip strength levels [77], combating one of the frailty criteria [13]. In the present study, MPA and grip strength were identified by the unadjusted and the adjusted regression analyses as potentially protective factors for DEP symptomatology.

Physical exercise practices have different underlying mechanisms (i.e., neurobiological, behavioral) that can promote physical and mental health [78,79]. In a current population-based study (n = 4052) carried out with individuals aged 24–105 years, fatigue, gait speed, self-reported PA (last two weeks), and inflammation (interleukin-6 and C-reactive protein high sensitivity) were evaluated [80]. The findings showed that reducing fatigue and inflammation and increasing PA levels can delay functional decline. In the group of men, in the third line of the decision tree, among those who presented fatigue but not slowness, handgrip strength was revealed as the strongest predictor of DEP symptomatology. On the other hand, among women, in the same line of the decision tree, weight loss was identified as the strongest predictor of DEP. Weight loss and grip strength were also suggested for both men and women on the other side of the tree (i.e., older adults with fatigue and no slowness) as strong predictors of DEP. These results suggested that DEP may be present even in older adults with preserved mobility and can be identified and treated.

### Strengths, Limitations, and Future Directions

The present study has limitations and strengths that must be considered. First, our findings may not be generalizable to groups outside the European continent. Second, four independent variables were obtained through self-reporting, which may not have accurately reflected these variables’ actual and absolute levels. Third, although age was controlled in the main analyses, we do not rule out that age-related memory problems may have influenced participants’ responses during the interviews. Fourth, there was a lack of control over the number of different types of medication in the analyses. This was not possible because the SHARE database did not provide drug information. The combination of different types of medication, including antidepressants, can cause interactions that affect different systems, affecting the health of the older frail-depressed [5]. Finally, it must be considered that the slowness variable presented by SHARE-FI and used in the present study to compose the frailty index does not fully represent the criterion exposed by Fried [14], which was targeted at physical or functional capacity. On the other hand, the present study has strengths: First, a longitudinal design established the temporal relationship between frailty and DEP symptomatology 5 years later. Second, including a large sample of older adults from various countries provides good external validity. Third, we adjusted the analyses for a wide range of potentially confounding factors associated with frailty that represent risk factors for DEP symptomatology. Fourth, through initial analyses it was verified that the exclusion of individuals with DEP symptomatology at baseline did not affect the longitudinal results (reverse causality). Finally, the findings provided detailed information of sex on frailty in older depressed individuals.

Suggestions for future investigations: (1) focus on the inter- and intra-individual differences that men and women present, which, in turn, influence the process of frailty and DEP symptomatology from age 50 onwards. Therefore, it would be important for investigations to look for behavioral and biological markers that consider the peculiarities of human aging; (2) expand and qualify the understanding of strategies based on physical exercise capable of increasing PA levels in the European older adult population, as a possible strategy to mitigate DEP symptomatology and frailty criteria [81]; (3) researchers should keep in mind the multicomponent arrangement of the human organism and that the coordination of all functions occur through an integrated system. From this perspective, it is important that strategies are not reduced to a single physiological system, but rather incorporate holistic principles [6], and seek to differentiate and specify interventions according to frail and non-frail older adults. The treatment of DEP and the criteria that make frailty require the appreciation of complex relationships that occur between and within metabolic systems in response to disruptive stress [13]; (4) it is also recommended that future studies explore uni- or bidirectional causal relationships between frailty and DEP symptomatology (including predictive and protective relationships) in the older population [6]; (5) we suggest that studies focus on the influence of DEP symptomatology on future frailty; in particular, how DEP could lead to inactivity, responsible for reduced handgrip strength and slowness. In advanced age, both are responsible for increasing the risk of deprivation of daily life activities, including social exclusion and loneliness; and (6) investigations with longitudinal follow-ups are also important to strengthen the understanding of the burden of multisystem dysregulation responsible for the increased risk of frailty and DEP symptomatology over the years [82].

## 5. Conclusions

Regardless of sex, for older adults aged 50 or over in 12 European countries, our longitudinal analysis pointed to fatigue, weight loss, and slowness as the main factors responsible for the increased risk of DEP 5 years later. On the other hand, greater MPA and grip strength were presented as possible protective factors against the risk of developing DEP symptomatology. According to the decision tree analysis, the prevalence of DEP among men was found in those with greater fatigue, weight loss, and slowness. In women, lower MPA was the third determining factor for the onset of DEP. According to our findings, possible strategies to reverse the factors associated with increased risk of DEP are linked to physical health: improving MPA intensity and muscle strength. From this, political decision-makers and health professionals can improve current actions aimed at reducing and combating frailty and DEP symptomatology in European citizens aged 50 and over.

## Figures and Tables

**Table 1:** Descriptive analysis of the variables studied (wave 6).

Variable	Total (n = 27,122)	Men (n = 11,688)	Women (n = 15,434)	*p*-Value
Age (years)				<0.001 *
Total (mean ± SD)	66.78 ± 8.86	67.03 ± 8.66	66.58 ± 9.00	
50–59 n (%)	6333 (23.3)	2536 (21.7)	3797 (24.6)	
60–69 n (%)	10,792 (39.8)	4757 (40.7)	6035 (39.1)	
70–79 n (%)	7524 (27.8)	3342 (28.6)	4182 (27.1)	
80–89 n (%)	2332 (8.6)	1005 (8.6)	1327 (8.6)	
90–99 n (%)	138 (0.5)	46 (0.4)	92 (0.6)	
Education n (%)				<0.001 ^†^
ISCED 0–1	7133 (26.3)	2641 (22.6)	4491 (29.1)	
ISCED 2–4	13,968 (51.5)	6078 (52.0)	7887 (51.1)	
ISCED 5–6	6021 (22.2)	2969 (25.4)	3056 (19.8)	
BMI				<0.001 *
Total (mean ± SD)	27.12 ± 4.57	27.43 ± 4.04	26.88 ± 4.92	
Underweight n (%)	219 (0.8)	28 (0.2)	191 (1.2)	
Normal n (%)	9462 (34.8)	3320 (28.4)	6142 (39.8)	
Overweight n (%)	11,372 (41.9)	5782 (49.5)	5590 (36.2)	
Obese n (%)	6069 (22.4)	2558 (21.9)	3511 (22.7)	
Living with partner				<0.001 ^†^
Yes n (%)	19,998 (73.7)	9750 (83.4)	10,248 (66.4)	
No n (%)	7124 (26.3)	1938 (16.6)	5186 (33.6)	
Chronic disease n (%)				<0.001 ^†^
0	6636 (24.5)	3053 (26.1)	3582 (23.2)	
1–3	17,370 (64.0)	7503 (64.2)	9867 (63.9)	
4–6	2926 (10.8)	1070 (9.2)	1856 (12.0)	
7–9	182 (0.6)	58 (0.4)	124 (0.7)	
10–14	8 (0.1)	4 (0.1)	4 (0.1)	
Disability n (%)				0.432 ^†^
Yes n (%)	2468 (9.1)	1075 (9.2)	1404 (9.1)	
No n (%)	24,654 (90.9)	10,613 (90.8)	14,030 (90.9)	
DEP				<0.001 ^†^
Total (mean ± SD)	2.22 ± 2.10	1.75 ± 1.84	2.57 ± 2.20	
No depressed n (%)	20,745 (76.5)	9835 (84.1)	10,958 (70.9)	
Depressed n (%)	6282 (23.2)	1806 (15.5)	4476 (29.0)	
Frailty				<0.001 ^†^
Non-frail n (%)	21,899 (80.7)	10,455 (89.4)	11,444 (67.6)	
Pre-frail n (%)	3923 (14.5)	1044 (8.9)	2879 (21.6)	
Frail n (%)	1300 (4.8)	189 (1.6)	1111 (10.8)	
Fatigue				<0.001 ^†^
Yes n (%)	9070 (33.4)	3236 (27.7)	5834 (37.8)	
No n (%)	18,052 (66.6)	8452 (72.3)	9600 (62.2)	
Weight loss				<0.001 ^†^
Yes n (%)	1951 (7.2)	618 (5.3)	1333 (8.6)	
No n (%)	25,171 (92.8)	11,070 (94.7)	14,101 (91.4)	
Slowness				<0.001 ^†^
Yes n (%)	3294 (12.1)	1080 (9.2)	2214 (14.3)	
No n (%)	23,828 (87.9)	10,608 (90.8)	13,220 (85.7)	
MPA				<0.001 *
Total (mean ± SD)	2.62 ± 11.54	2.65 ± 0.66	2.60 ± 0.70	
<Once week n (%)	3277 (12.1)	1274 (10.9)	2004 (13.0)	
Once week n (%)	3642 (13.4)	1540 (13.2)	2102 (13.6)	
>Once week n (%)	20,203 (74.5)	8875 (75.9)	11,328 (73.4)	
Grip strength (kg)				
Total (mean ± SD)	34.26 ± 11.19	43.93 ± 9.44	26.94 ± 6.49	<0.001 *

BMI: body mass index; DEP: depression; MPA: moderate physical activity; SD: standard deviation; ^†^ Chi-square test *p* < 0.001; * Mann-Whitney U test *p* < 0.001.

**Table 2:** Results of the association between frailty (wave 6) and depression (wave 8), according to sex.

Variable	Unadjusted OR (95% CI)	*p*-Value	Adjusted OR (95% CI)	*p*-Value
Men				
Slowness	0.470 (0.405, 0.545)	<0.001	0.552 (0.478, 0.649)	<0.001
Weight loss	0.558 (0.472, 0.678)	<0.001	0.582 (0.488, 0.702)	<0.001
Fatigue	0.346 (0.315, 0.387)	<0.001	0.369 (0.335, 0.413)	<0.001
MPA	−0.910 (0.852, 0.983)	<0.001	−0.901 (0.844, 0.976)	<0.001
Grip strength	−0.978 (0.975, 0.985)	<0.001	−0.984 (0.982, 0.994)	<0.001
Women				
Slowness	0.562 (0.513, 0.632)	<0.001	0.690 (0.626, 0.779)	<0.001
Weight loss	0.578 (0.516, 0.661)	<0.001	0.588 (0.528, 0.678)	<0.001
Fatigue	0.391 (0.363, 0.422)	<0.001	0.421 (0.395, 0.461)	<0.001
MPA	−0.860 (0.822, 0.913)	<0.001	−0.872 (0.833, 0.926)	<0.001
Grip strength	−0.970 (0.965, 0.976)	<0.001	−0.970 (0.973, 0.985)	<0.001

**Table 3:** Gain index of predicting depression for men.

Node	Node		Gain		Response	Index
	N	%	N	%		
4	598	5.1%	301	14.2%	50.3%	277.4%
7	294	2.5%	121	5.7%	41.2%	226.8%
12	234	2.0%	83	3.9%	35.5%	195.5%
8	1142	9.8%	342	16.1%	29.9%	165.0%
10	215	1.8%	57	2.7%	26.5%	146.1%
9	1202	10.3%	310	14.6%	25.8%	142.1%
13	248	2.1%	46	2.2%	18.5%	102.2%
11	7755	66.4%	861	40.6%	11.1%	61.2%

Growing Method: CHAID.

**Table 4:** Gain index of predicting depression for women.

Node	Node		Gain		Response	Index
	N	%	N	%		
16	567	3.7%	379	8.2%	66.8%	222.4%
11	67	0.4%	37	0.8%	55.2%	183.7%
15	850	5.5%	463	10.0%	54.5%	181.2%
13	618	4.0%	335	7.2%	54.2%	180.3%
14	3799	24.6%	1472	31.7%	38.7%	128.9%
12	730	4.7%	256	5.5%	35.1%	116.7%
7	479	3.1%	151	3.3%	31.5%	104.9%
8	1442	9.3%	342	7.4%	23.7%	78.9%
9	2847	18.4%	553	11.9%	14.9%	64.6%
10	4035	26.1%	651	14.0%	16.1%	53.7%

Growing Method: CHAID.

## Data Availability

Data may be accessed through becoming a registered user with the Survey of Health, Ageing and Retirement in Europe (via www.share-project.org) (accessed on 22 November 2023).
